# Dynamic simulations on the mitochondrial fatty acid Beta-oxidation network

**DOI:** 10.1186/1752-0509-3-2

**Published:** 2009-01-06

**Authors:** Robert Modre-Osprian, Ingrid Osprian, Bernhard Tilg, Günter Schreier, Klaus M Weinberger, Armin Graber

**Affiliations:** 1 eHealth systems, Biomedical Engineering, Austrian Research Centers GmbH – ARC, Eduard Wallnoefer Zentrum 1, 6060 Hall in Tirol, Austria; 2 BIOCRATES life sciences AG, Innrain 66/2, 6020 Innsbruck, Austria; 3 Institute for Biomedical Engineering, UMIT, Eduard Wallnoefer Zentrum 1, 6060 Hall in Tirol, Austria; 4 Institute for Bioinformatics, UMIT, Eduard Wallnoefer Zentrum 1, 6060 Hall in Tirol, Austria

## Abstract

**Background:**

The oxidation of fatty acids in mitochondria plays an important role in energy metabolism and genetic disorders of this pathway may cause metabolic diseases. Enzyme deficiencies can block the metabolism at defined reactions in the mitochondrion and lead to accumulation of specific substrates causing severe clinical manifestations. Ten of the disorders directly affecting mitochondrial fatty acid oxidation have been well-defined, implicating episodic hypoketotic hypoglycemia provoked by catabolic stress, multiple organ failure, muscle weakness, or hypertrophic cardiomyopathy. Additionally, syndromes of severe maternal illness (HELLP syndrome and AFLP) have been associated with pregnancies carrying a fetus affected by fatty acid oxidation deficiencies. However, little is known about fatty acids kinetics, especially during fasting or exercise when the demand for fatty acid oxidation is increased (catabolic stress).

**Results:**

A computational kinetic network of 64 reactions with 91 compounds and 301 parameters was constructed to study dynamic properties of mitochondrial fatty acid β-oxidation. Various deficiencies of acyl-CoA dehydrogenase were simulated and verified with measured concentrations of indicative metabolites of screened newborns in Middle Europe and South Australia. The simulated accumulation of specific acyl-CoAs according to the investigated enzyme deficiencies are in agreement with experimental data and findings in literature. Investigation of the dynamic properties of the fatty acid β-oxidation reveals that the formation of acetyl-CoA – substrate for energy production – is highly impaired within the first hours of fasting corresponding to the rapid progress to coma within 1–2 hours. LCAD deficiency exhibits the highest accumulation of fatty acids along with marked increase of these substrates during catabolic stress and the lowest production rate of acetyl-CoA. These findings might confirm gestational loss to be the explanation that no human cases of LCAD deficiency have been described.

**Conclusion:**

In summary, this work provides a detailed kinetic model of mitochondrial metabolism with specific focus on fatty acid β-oxidation to simulate and predict the dynamic response of that metabolic network in the context of human disease. Our findings offer insight into the disease process (e.g. rapid progress to coma) and might confirm new explanations (no human cases of LCAD deficiency), which can hardly be obtained from experimental data alone.

## Background

**Figure 1:**
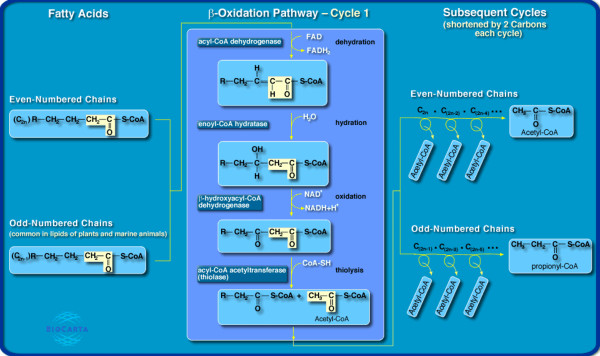
**β-Oxidation pathway of fatty acids (with permission of Biocarta)**. The β-oxidation cycle itself is catalyzed by a series of four enzymes in the mitochondrial matrix. Each turn of the cycle shortens the fatty acid chain by two carbon atoms and generates one molecule of acetyl CoA. We only consider even-chain saturated fatty acids.

Mitochondrial β-oxidation of fatty acids plays a major role in energy production, especially during periods of fasting or low intensity exercise. The primary sources of fatty acids for oxidation are dietary and mobilization of triacylglycerols mainly stored in adipocytes of adipose tissue. The release of metabolic energy, in the form of fatty acids, is controlled by a complex series of interrelated cascades that result in the activation of hormone-sensitive lipase, which hydrolyzes fatty acids from triacylglycerols and diacylglycerols. The final fatty acid is released from monoacylglycerols through the action of monoacylglycerol lipase, an enzyme active in the absence of hormonal stimulation. Once released, these fatty acids travel through the blood to other tissues such as muscle where they are oxidized to provide energy through the mitochondrial β-oxidation pathway. The β-oxidation spiral of fatty acid metabolism involves these four steps: oxidation, hydration, a second oxidation, and finally thiolysis. These occur in repeating cycles through the sequential removal of 2 carbons and production of acetyl-CoA, which then enters the Krebs cycle for oxidation and ATP production (Figure 1). Another destination of acetyl-CoA is the production of ketone bodies in the liver that are transported to tissues like the heart and brain for energy production during starvation. Fatty acids with an odd number of carbons in the acyl chain are left at the end with propionyl-CoA, which is converted to succinyl-CoA that then also enters the Krebs cycle. Furthermore, unsaturated fatty acids with bonds in the *cis *configuration require three separate enzymatic steps to prepare themselves for the β-oxidation pathway.

**Figure 2:**
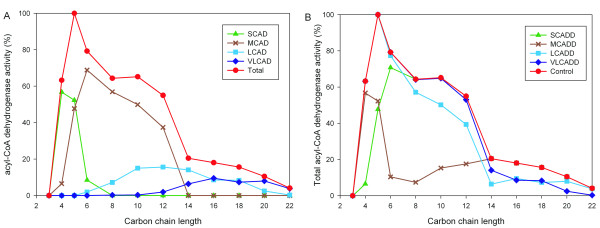
**Specific enzyme complex activity of acyl-CoA dehydrogenase deficiencies**. (A) Activity distribution of four distinct acyl-CoA dehydrogenases with respect to fatty acid carbon chain length based on data from literature [3-5]. Short-chain (SCAD), medium-chain (MCAD), long-chain (LCAD) and very long-chain (VLCAD) acyl-CoA dehydrogenase is defined according to the specific enzyme complex activity catalyzing dehydration of short-, medium-, long- and very long-chain fatty acids. The total activity of all four acyl-CoA dehydrogenases is shown in red (Total). (B) Total acyl-CoA dehydrogenase activity as a function of carbon chain length without deficiencies in enzyme activity (Control). Reduced activity of acyl-CoA dehydrogenases yields to different total activity reflecting deficiencies of short-chain (SCADD), medium-chain (MCADD), long-chain (LCADD) and very long-chain (VLCADD) acyl-CoA dehydrogenase, respectively.

Mitochondrial fatty acid oxidation deficiencies are due to genetic defects in enzymes of fatty acid β-oxidation and transport proteins (clinically often summarized as FATMO – fatty acid transport and mitochondrial oxidation). Genetic defects have been identified in most of the genes where nearly all types of sequence variations (mutation types) have been associated with disease [1]. In particular, defects in fatty acid metabolism associated with clinical disorders include defects in acyl-CoA dehydrogenase and β-hydroxyacyl-CoA dehydrogenase, which catalyzes the first and third steps in β-oxidation, respectively. Several acyl-CoA dehydrogenases were previously isolated and described [2-6]. In general, these enzymes can be classified due to their fatty acid chain length specificity in short-chain (SCAD), medium-chain (MCAD), long-chain (LCAD), and very long-chain acyl-CoA dehydrogenases (VLCAD). The enzymatic activity distributions of the fatty acid chain length specificity of these four enzymes overlap to some extent [2-4,7], as summarized in Figure 2A. Consequently, the deficiency of one particular enzyme cannot be compensated by the accumulated activity of the other non-impaired enzymes (Figure 2B).

Ten of the disorders directly affecting mitochondrial fatty acid oxidation have been well-defined, implicating episodic hypoketotic hypoglycemia provoked by catabolic stress, multiple organ failure, muscle weakness, or hypertrophic cardiomyopathy. Additionally, syndromes of severe maternal illness (HELLP syndrome and AFLP) have been associated with pregnancies carrying a fetus affected by fatty acid oxidation deficiencies. The incidence of one of these disorders, MCAD deficiency (MIM 201450), is 1:14 600 in almost 8.2 million newborns worldwide [8]. In the first years of life this inherited deficiency may become apparent following a prolonged fasting period, sometimes in combination with infection or fever. An acute attack usually features symptoms of lethargy, nausea and vomiting, which rapidly progresses to coma within 1–2 h. Up to 25% of MCAD patients die during their first attack; or suffer permanent brain damage from cerebral edema. The clinical phenotypes of most of the disorders of fatty acid metabolism are very similar [9].

The introduction of tandem mass spectrometry (MS/MS) for the analysis of plasma acylcarnitines has greatly facilitated the identification of patients with a defect in fatty acid β-oxidation and has unquestionably been the most striking recent advance in newborn screening. Pre-symptomatic diagnosis is important to prevent morbidity as most of the diagnosed defects are treatable and the prognosis is generally favorable [10-12].

Besides statistical model building and data mining based approaches [13-15], computational Systems Biology is essential to combine knowledge of human physiology and pathology starting from genomics, molecular biology, and the environment through the levels of cells, tissues, and organs all the way up to integrated systems behavior. Applying Systems Biology approaches within the context of human health and disease will definitely gain new insights. Eventually, a new discipline – Systems Medicine – will emerge at the interface between Medicine and Systems Biology [16-18]. Higher levels of organization are extremely complex and even models at the cell and subcellular levels are forced to resort to simplifications to minimize modeling and computational complexity [19-21]. Additionally, some parameters and constants for kinetics, binding and concentrations of biomolecules are typically not known, thus reducing the model's ability to respond correctly to dynamic changes in external conditions. A high-quality network of human-specific metabolic pathways including detailed knowledge about all metabolic reactions concerned is essential to design tailored kinetic models for better understanding of human metabolism and its relationship with diseases. While such large networks are used to analyze the global structure or functional connectivity of the network [22], deterministic and stochastic models are mainly used for simulating specific metabolic pathways as well as regulatory and signaling networks [23].

To date, little is known about fatty acids kinetics, especially during catabolic stress or exercise when the demand for fatty acid oxidation is increased. Here we introduce a detailed kinetic model of mitochondrial metabolism with specific focus on fatty acid β-oxidation to simulate and predict the dynamic response of that metabolic network toward distinct enzyme deficiencies. The simulation results are compared and validated using experimental data. Finally, the dynamic response to changes in the input to the system representing catabolic stress is simulated and results are interpreted in a biological and clinical context, followed by a discussion on limitations of the model.

## Results

### Construction and evaluation of kinetic model

**Figure 3:**
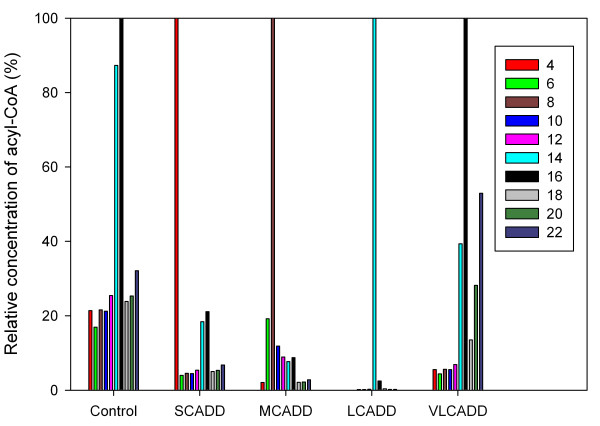
**Simulated acyl-CoA concentrations**. Simulated relative concentration of acyl-CoA as a function of carbon chain length for healthy controls (Control) and acyl-CoA dehydrogenase deficiencies (SCADD, MCADD, LCADD, VLCADD). The concentrations of each deficiency are divided by the respective maximum value of concentration. The simulations show accumulation of acyl-CoAs with a maximum at carbon chain lengths of 4 (SCADD), 8 (MCADD), 14 (LCADD) and 16 (VLCAD, Control).

The major objectives of this study were to construct a dynamic simulation environment allowing the exploration of complex biochemical processes involved in fatty acid β-oxidation, the validation of the model with experimental data, and finally the application of these pathway models to the analysis of metabolic diseases. We therefore built a deterministic model describing the biochemical reactions and pathway in the form of kinetic rate equations, and investigated the dynamic response of the system to specific perturbations of enzyme activities. Based on a publicly available computational model for mitochondrial metabolism (see the methods section) and previously described enzymatic activity distributions (Figure 2A, B), we were able to build a detailed kinetic model of the mitochondrial β-oxidation, which allows to simulate and analyze acyl-CoA dehydrogenase deficiencies. The simulated concentrations of acyl-CoAs are shown in Figure 3. In healthy controls the maximum value was observed for C16, followed by C14 reflecting the distribution of fatty acids entering the β-oxidation cycle with its maximum at C16.

The reduced activity of acyl-CoA dehydrogenases leads to a deviation of the total activity as compared to healthy controls (Figure 2B) and, subsequently, to the accumulation of specific acyl-CoAs (Figure 3). For example, the reduced acyl-CoA dehydrogenase activity for fatty acids with carbon chain lengths of 4 and 6 results mainly in an increase of acyl-CoAs with carbon chain length 4 in case of SCAD deficiency. The low enzyme activity in MCAD deficiency at chain lengths of 6 to 12 is reflected by a high concentration of octanoyl-CoA (C8). The model predicts the accumulation of specific acyl-CoAs corresponding to the investigated enzyme deficiencies which are in agreement with findings in the literature [24].

### Relationship between model and experimental data

Isotope-dilution MS/MS on plasma or whole blood facilitates the measurements of acylcarnitines and the diagnosis of newborns with a defect in fatty acid β-oxidation [25,26]. The transport of fatty acyl-CoA into the mitochondria is accomplished via an acylcarnitine intermediate generated through trans-esterification of the fatty acid moiety from CoA to carnitine by carnitine palmitoyl transferase I (CPT I). The acylcarnitine molecules are then transported across the organelle's inner membrane into the mitochondrial matrix by carnitine acylcarnitine translocase where the re-esterification of the fatty acyl-CoA molecule and, eventually, β-oxidation occurs. The diagnosis of fatty acid β-oxidation disorders is based on the assumption that there is an association between the accumulations of specific chain length acylcarnitines in the mitochondria with the deficiency of a distinct acyl-CoA dehydrogenase in the mitochondrial matrix [27]. Amino acid, carnitine and acylcarnitine concentrations were documented in newborn screening programs in Middle Europe and South Australia [28,29]. A summary of regional dissimilarities of acylcarnitines (with even-chain saturated fatty acid acyl group) concentrations is provided in Table 1.

**Table 1:** Acylcarnitine concentrations of screened newborns

**Acylcarnitines [μmol/l]**			**C4**	**C6**	**C8**	**C10**	**C12**	**C14**	**C16**	**C18**
**Middle Europe**	**healthy controls (n = 590.216)**	**median**	0.38	0.12	0.10	0.09	0.13	0.21	4.37	0.97
		
		**IQR**	0.27	0.14	0.07	0.09	0.09	0.13	2.18	0.52
	
	**MCAD deficiency (n = 63)**	**median**	0.40	1.31	5.14	0.59	0.11	0.19	3.47	0.80
		
		**IQR**	0.23	1.27	8.78	0.64	0.10	0.12	1.59	0.46
	
	**VLCAD deficiency (n = 5)**	**median**	0.28	0.11	0.12	0.15	0.47	0.85	3.15	1.38
		
		**IQR**	0.11	0.07	0.08	0.13	0.19	0.45	1.04	1.44

**South Australia**	**MCAD deficiency (n = 13)**	**median**	0.32	0.97	7.23	1.10	0.25	0.32	3.49	2.69
		
		**IQR**	0.07	0.95	7.27	0.39	0.09	0.13	1.80	1.18
	
	**VLCAD deficiency (n = 3)**	**median**	0.40	0.16	0.15	0.34	1.19	2.41	4.99	3.11
		
		**IQR**	0.27	0.08	0.10	0.21	0.42	2.71	3.22	5.43

Summary of regional dissimilarities of acylcarnitines (with even-chain saturated fatty acid acyl group) concentrations (median and interquartile range (IQR)) of newborns screened in Middle Europe and South Australia [28,29].

**Figure 4:**
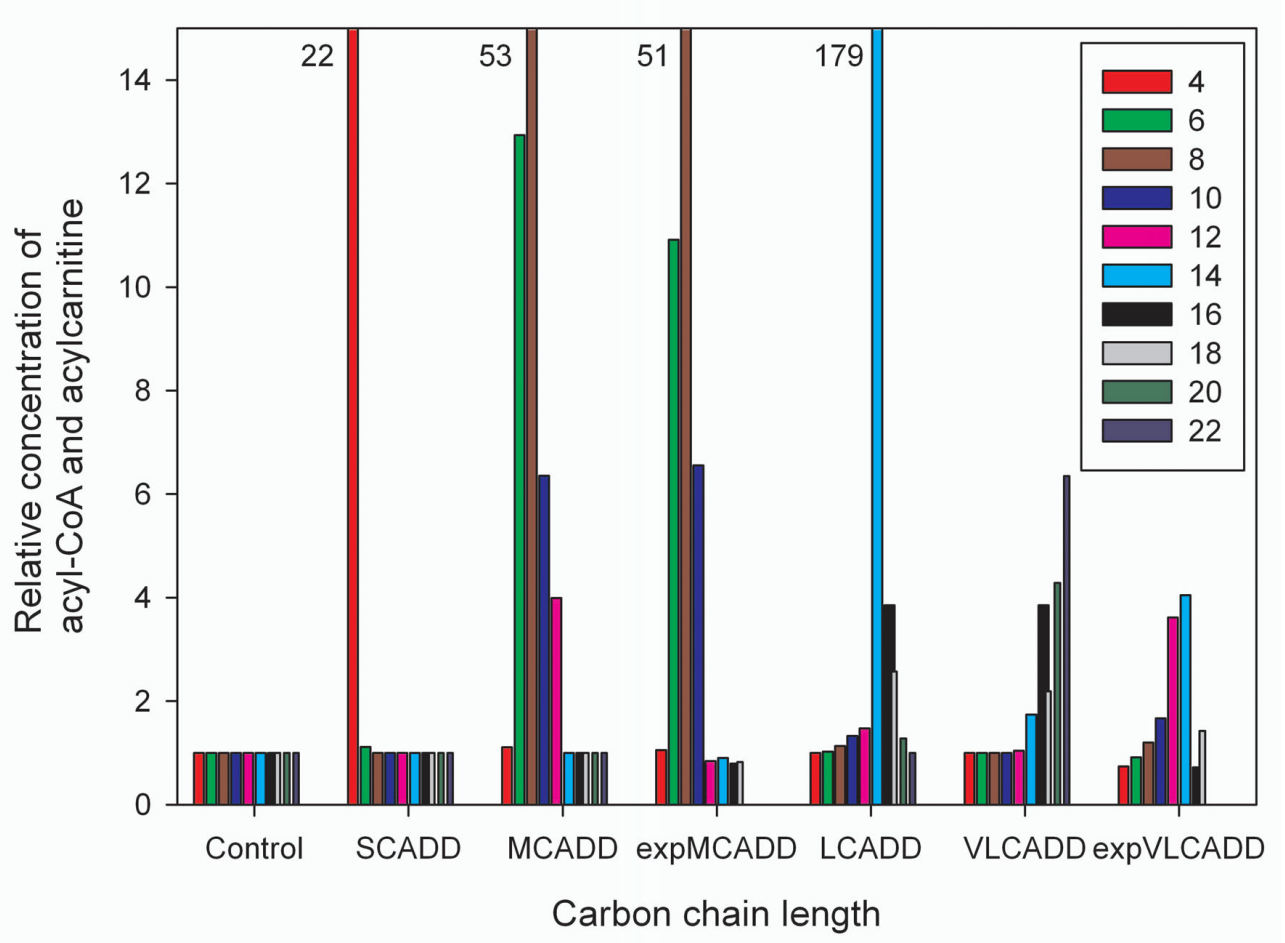
**Simulated acyl-CoA concentrations and experimentally derived acylcarnitine data**. Relative simulated concentration of acyl-CoA as a function of carbon chain length for healthy controls (Control) and acyl-CoA dehydrogenase deficiencies (SCADD, MCADD, LCADD, VLCADD). The concentrations are divided by the concentrations of the control group (Controls). Additionally, experimentally derived acylcarnitine data for C4 to C18 of MCAD (expMCADD) and VLCAD (expVLCADD) deficiencies of the Middle Europe dataset are depicted. These data are divided by the concentrations of the corresponding experimental control data. Note that relative concentration values higher than 14 are shown on the top x-axis at carbon lengths of 4, 8 and 14. The experimental data are unlinked anonymous newborn screening data from Germany [28].

In order to compare the data from the screening programs to accumulating fatty acyl-CoA concentrations for the simulated enzyme deficiencies, we calculated relative concentrations with respect to the simulated control group (Controls) and healthy control from the screening programs, respectively. We did not directly compare acyl-CoA in the mitochondria with measured acylcarnitine concentrations outside the mitochondria. Based on diagnostic findings and conclusions in newborn screening programs we assumed similar relative behavior of acylcarnitines and acyl-CoAs. Simulation revealed that LCAD deficiency showed the largest effect of acyl-CoA accumulation (179-fold), followed by MCAD deficiency (53-fold) corresponding well with experimentally derived MCAD deficiency data (51-fold) of the Middle Europe data set (Figure 4). A less strong effect (22-fold increase) was observed on simulation data of SCAD deficiency. The experimental and simulated VLCAD deficiency data differ from each other. Simulations demonstrated an accumulation of C14 and C16 as well as C18 to C22 while experimental data indicated accumulation of C10 to C14 with hardly any effect on C16 (experimental data of C20 and C22 were not available).

We additionally provide acylcarnitine ratios with respect to C4 concentration on experimentally derived data of newborn screening programs in Middle Europe and South Australia as well as simulation data with different C16 input (Simulation A and B, see the methods section) in Table 2 and Table 3. The overall profile of the ratios matches well, showing the highest ratio at C8/C4 for MCAD deficiency (MCADD) and C14/C4 for VLCAD deficiency (VLCADD).

**Table 2:** MCADD ratios of model and experimentally derived data

**MCADD**	**C6/C4**	**C8/C4**	**C10/C4**	**C12/C4**	**C14/C4**
**Middle Europe**	3.28	12.85	1.48	0.28	0.48

**South Australia**	3.03	22.59	3.44	0.78	1.00

**Simulation A**	9.22	47.97	5.68	4.28	3.67

**Simulation B**	9.82	90.55	5.97	4.43	3.78

C6/C4, C8/C4, C10/C4, C12/C4 and C14/C4 ratios for MCADD of acylcarnitine concentrations of experimentally derived data. Similar ratios of acyl-CoA concentrations are given for the simulation data.

**Table 3:** VLCADD ratios of model and experimentally derived data

**VLCADD**	**C6/C4**	**C8/C4**	**C10/C4**	**C12/C4**	**C14/C4**
**Middle Europe**	0.39	0.43	0.54	1.68	3.04

**South Australia**	0.40	0.38	0.85	2.98	6.03

**Simulation A**	0.79	1.01	0.99	1.24	7.09

**Simulation B**	0.79	1.01	0.99	1.24	7.51

C6/C4, C8/C4, C10/C4, C12/C4 and C14/C4 ratios for VLCADD of acylcarnitine concentrations of experimentally derived data. Similar ratios of acyl-CoA concentrations are given for the simulation data.

### Responses of the model to dynamic changes

**Figure 5:**
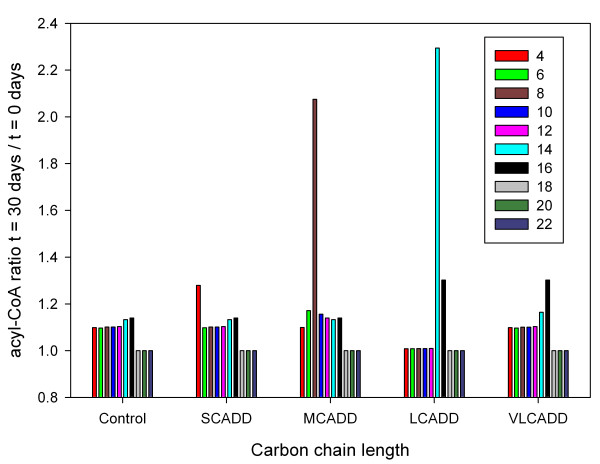
**Ratios of simulated acyl-CoA concentrations before and after fasting**. Ratios of simulated acyl-CoA concentrations before and 30 days after a 20% increase of palmitoyl-CoA (C16). The dynamic change leads to more than two-fold increases of octanoyl-CoA (C8) concentrations for MCAD deficiency (MCADD) and tetradecanoyl-CoA (C14) for LCAD deficiency (LCADD), respectively.

Clinical manifestation of MCAD deficiency usually starts after significant catabolic stress. When carbohydrate stores are depleted, the organism switches to energy production from stored triacylglycerols, which results in lipolysis and release of fatty acids. In MCAD deficiency a dramatic rise of plasma levels of specific free fatty acids is observable, indicating impaired β-oxidation of respective chain-length acyl-CoA. Additionally, ketones remain inappropriately low, reflecting the defect in hepatic fatty acid oxidation. Hypoglycemia develops shortly thereafter, probably because of excessive glucose utilization due to the inability to switch to fat as a fuel [9]. Thus, we simulated the consequences of the mobilization of fatty acids induced by fasting leading to increased acyl-CoA concentrations for fatty acid β-oxidation by increasing palmitoyl-CoA (C16). An increase of about 20% showed more than two-fold increases of octanoyl-CoA (C8) concentrations for the deficiency MCAD and tetradecanoyl-CoA (C14) for the deficiency LCAD, respectively (Figure 5). A lesser effect was observed for the deficiencies SCAD and VLCAD. A higher increase of palmitoyl-CoA (C16) resulted in higher accumulation of specific acyl-CoA concentrations. In Table 4 ratios of specific acyl-CoA concentrations before and 30 days after 20%, 30%, and 40% increase of palmitoyl-CoA (C16) are given.

**Table 4:** Ratios of simulated acyl-CoA concentrations before and after fasting

	**20%**	**30%**	**40%**
**Control C8-ratio**	1.10	1.15	1.19

**SCADD C4-ratio**	1.28	1.44	1.62

**MCADD C8-ratio**	2.08	3.59	6.97

**LCADD C14-ratio**	2.29	2.89	3.46

**VLCADD C14-ratio**	1.16	1.25	1.33

Ratios of simulated acyl-CoA concentrations before and 30 days after 20%, 30%, and 40% increase of palmitoyl-CoA (C16). Note that the time to attain the steady-state increases with higher increase of C16 and is in case of LCADD higher than 30 days for 30% and 40% increase of C16 resulting in lower ratios (2.89 and 3.46) than the ratios calculated for MCADD (3.59 and 6.97).

**Figure 6:**
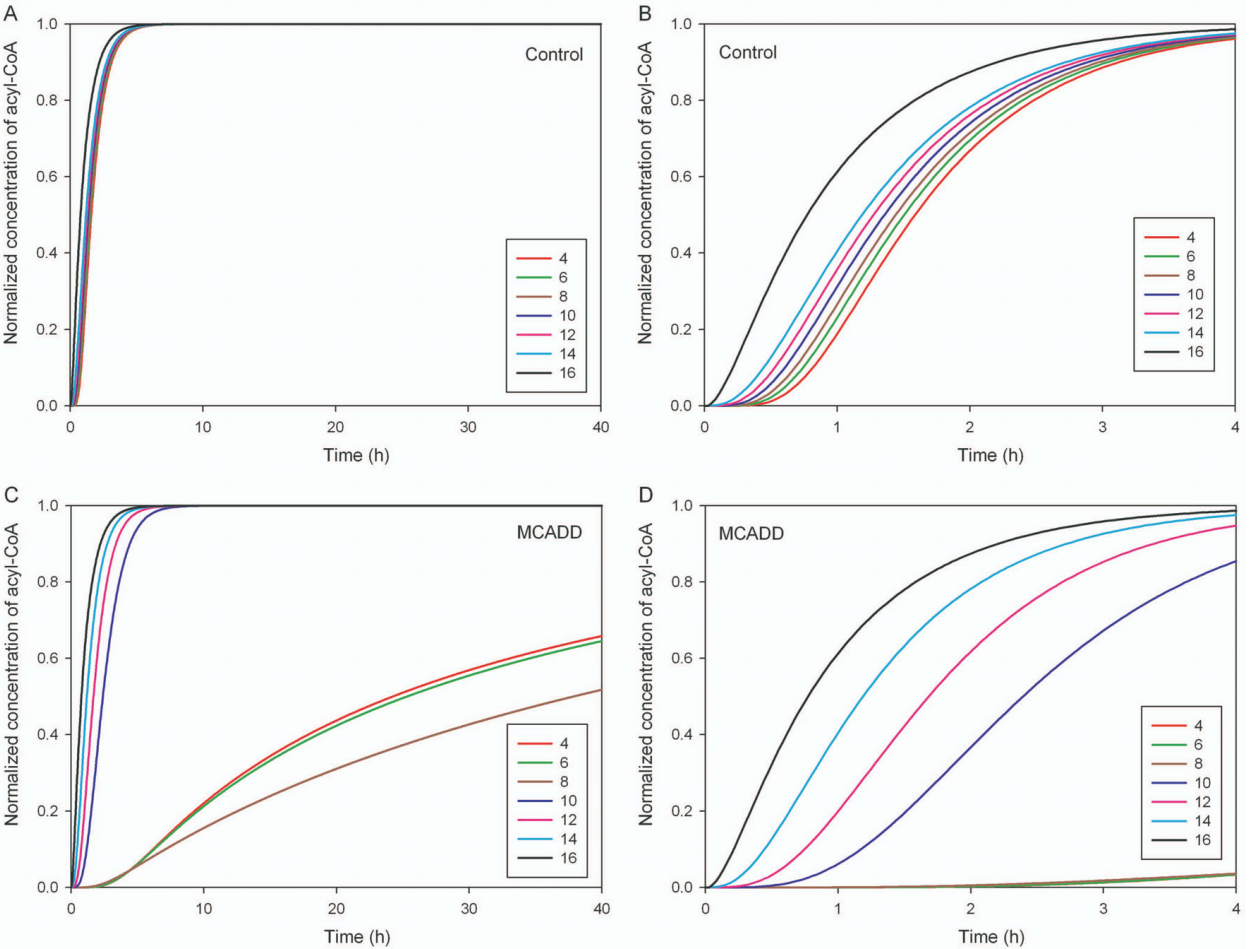
**Simulated dynamic acyl-CoA concentration during fasting**. (A, B) Dynamic change of simulated concentrations of acyl-CoA of different carbon chain length in healthy controls following a 20% increase of palmitoyl-CoA (C16) concentration. Concentration values were shifted in terms of their minima and normalized with respect to their maximum change resulting in normalized concentration values between 0 and 1. After 4 hours almost all acyl-CoAs reached their new steady-state values. (C, D) A 20% increase needs much longer (10 days) to attain the steady-state in the MCAD deficiency simulation.

Next we studied the repeating cycles of the β-oxidation spiral of fatty acid metabolism that sequentially removes 2 carbons. After the palmitoyl-CoA (C16) concentration was increased by about 20%, acyl-CoA concentrations reached a steady-state after 6 hours in healthy controls (Figure 6A). The depicted concentration values were shifted in terms of their minima and normalized with respect to their maximum change resulting in normalized concentration values between 0 and 1. The increase of the input to the β-oxidation cycle subsequently increased acyl-CoAs along the cascade from C16 to C4 with a delay of approximately one hour at a normalized concentration of 0.5 (Figure 6B). In contrast, a similar increase needed almost 10 days to attain equivalent steady-state in the MCAD deficiency simulation (Figure 6C). Due to inadequate enzymatic activity, medium-chain acyl-CoAs accumulate leading to a slow enzymatic clearance, specifically of octanoyl-CoA (C8). While C16 to C10 were subsequently increased with delay twice as long as healthy controls (Figure 6D), the subsequent increase of C8 to C4 switched resulting in a delay for C8 of 40 hours at a normalized concentration of 0.5 (Figure 6C). The same characteristic were found when increasing the palmitoyl-CoA (C16) concentration by 30% and 40% (data not shown).

**Figure 7:**
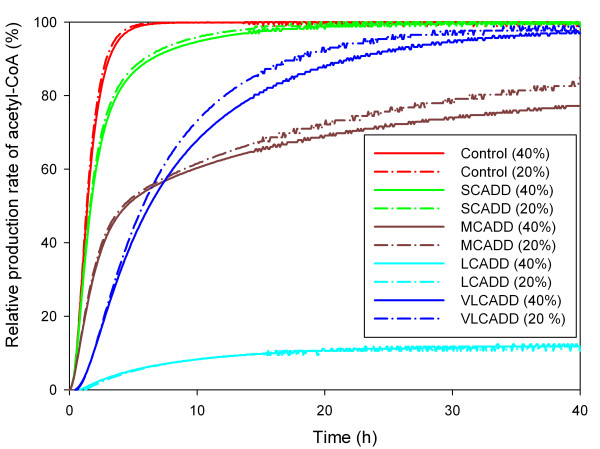
**Simulated production of acetyl-CoA during fasting**. Relative production rate of acetyl-CoA as a function of time following a 20% and 40% increase of palmitoyl-CoA (C16) concentration. After 4 hours the production rate of acetyl-CoA of healthy controls reached its maximum. In the case of simulating MCAD deficiency, the production rate of acetyl-CoA is still 50% below its maximum after 4 hours; whereas simulations of the LCAD deficiency showed a very low production rate of acetyl-CoA even after 40 hours – reaching about 10% of the production rate of healthy controls.

Acetyl-CoA – the final product of the β-oxidation spiral – is required for the production of energy and ketone bodies, especially during periods of fasting. Deficiencies of acyl-CoA dehydrogenases resulted in reduced production of acetyl-CoA. Following a 20% increase of palmitoyl-CoA (C16) concentration, the highest shortage of 30 days acetyl-CoA production was found in LCAD deficiency. The acetyl-CoA generated by the β-oxidation within 30 days was reduced by 6.3% compared to acetyl-CoA generated in the healthy situation. Figure 7 depicts the relative production rate of acetyl-CoA as a function of time following a 20% and 40% increase of C16. After 4 hours the production rate of acetyl-CoA of healthy controls reached its maximum. In the case of simulating MCAD deficiency, the production rate of acetyl-CoA is still 50% below its maximum after 4 hours; whereas simulations of the LCAD deficiency showed a very low production rate of acetyl-CoA even after 40 hours – reaching about 10% of the production rate of healthy controls.

## Discussion

The present computer simulation attempts to contribute to a better understanding (explanation) of pathophysiological aspects of a group of hereditary disorders impairing mitochondrial β-oxidation. Several enzymes are involved in mitochondrial fatty acid oxidation. For all of them, genetic defects impairing their function have been described. The availability of modern analytical methods has facilitated newborn screening for these disorders. For the identification of patients with a defect in β-oxidation, acylcarnitines in blood were evaluated showing a characteristic profile depending on the affected enzyme even in asymptomatic stage. We simulated the steady-state concentrations of acyl-CoAs in acyl-CoA dehydrogenase deficiencies and compared the results to acylcarnitine data from screening programs in Middle Europe and South Australia. Results indicate that the overall characteristics of the simulated accumulation of acyl-CoA show good agreement with experimental data and findings in the literature (Figure 3 and Figure 4).

Differences in simulated C16 acyl-CoA and measured C16 acylcarnitine might be caused by the special biological role of C16 acylcarnitine. From a biochemical perspective it is not clearly evident why a decrease of acyl-CoA dehydrogenase activity (Figure 2B) would not impact the concentration of C16 acyl-CoA in the mitochondria, leading to a much higher C16 acyl-CoA than C14 acyl-CoA concentration, which is reflected by the measured acylcarnitines (Table 1). It seems that there is an abundant pool of C16 acylcarnitine in the blood, whose level is almost not affected by mitochondrial fatty acid oxidation deficiencies (as can be seen in Table 1). This might be reasonable, since C16 acylcarnitine is an ester of an important saturated fatty acid, which is involved in several biological processes within the body – it is needed for energy, hormone production, cellular membranes and for organ padding as well as for important signaling and stabilization processes in the body.

Differences in simulated acyl-CoA ratios and measured acylcarnitine ratios shown in Table 2 are mainly given by the relatively small value of the simulated C4 acyl-CoA concentration and the relatively large value of the C12 acyl-CoA concentration. Higher values for C4 and lower values for C12 can be mainly generated by changing the activity of the enzymes for chain length 4 and 12. The problem is that we do not exactly know the total enzyme activity because the enzyme activities are based on data from rat liver mitochondria [3-5] (and not on data from human mitochondria). Additionally, mitochondria from different tissues can show different total acyl-CoA dehydrogenase activity as well as possible residual activities of the deficient enzyme are also changing the total acyl-CoA dehydrogenase activity of the individual patient. This might be supported by the high inter-patient variance of the measured acylcarnitines, which is caused not only by the experimental error but also by the biological variance between patients. For example, for some patient data the C12 acylcarnitine concentration is higher than the C14 acylcarnitine concentration (data are not shown). The discrepancy with C14 and C12 (for the simulation data the C12 ratio is higher than the C14 ratio which was not found in the experimental data) can be considered negligible, because of the small difference between C14 and C12 compared to the strong increase of C8 for MCADD. The ratio C8/C14 is more than 10 fold higher than the ratio C12/C14.

The clinical phenotypes of most of the disorders are very similar. As MCAD deficiency is the most prevalent defect among them we focused our discussion and biochemical interpretation on this particular defect. Nevertheless, the aspects are relevant to the pathogenesis of all fatty acid oxidation defects. Patients with MCAD deficiency are without clinical manifestations until a prolonged fasting period sometimes in combination with infection or fever. As a physiological response to this catabolic stress triacylglycerols from adipose tissue are released and energy production switches from carbohydrate to lipid utilization. In healthy individuals, subsequently ketone body formation by the liver is increased to provide this metabolic fuel for brain and muscles.

The response to catabolic stress in patients with MCAD deficiency shows a marked increase in plasma fatty acids, mitochondrial acylcarnitines and acyl-CoAs. Severe symptoms of lethargy and nausea develop as a consequence of encephalopathy, and patients can become dangerously ill, sometimes before plasma glucose falls to hypoglycemic levels. The progression to severe sickness proceeds within a few hours. Patients often die in the course of the first episode or at least suffer from persistent brain damage. The underlying pathogenetic mechanisms have been poorly understood until now. To simulate the response to fasting we assumed a 20% increase of palmitoyl CoA (C16) and observed significant differences of the calculated metabolic changes in acyl-CoA deficiencies with respect to the healthy controls (Figure 5). One major consequence of the disorder is inadequate ketone body formation to meet tissue energy demands under conditions of fasting and catabolic stress. Our calculations showed that formation of acetyl-CoA – substrate for energy production via the tricarboxylic acid (TCA) cycle and ketogenesis – is impaired (Figure 7). The simulation impressively showed the low production rate of acetyl-CoA within the first hours, which corresponds to the rapid disease progression after onset. In addition, inadequate acetyl-CoA production has secondary effects on flux through the TCA cycle, on regulation of fatty acid oxidation, and on efficiency of gluconeogenesis, which contribute to pathogenesis [24].

The simulated accumulation of specific acyl-CoAs according to the investigated enzyme deficiencies are in agreement with the accumulation of plasma free fatty acid intermediates, which enter the central nervous system and exert toxic effects, which may explain the observed encephalopathy and cerebral edema. In vitro experiments on cerebral cortex of rats indicate that inhibition of energy metabolism and oxidative stress induction by the accumulating fatty acids may contribute to the pathophysiology of encephalopathy [30,31].

Although several patients have been found to have VLCAD deficiency, none have been documented with LCAD deficiency [32]. This could arise from either gestational loss due to LCAD deficiency as seen in the mouse model, a failure to recognize LCAD deficiency because the phenotype differs so greatly from other inborn errors of fatty acid metabolism, or absence of disease resulting from LCAD deficiency in humans [33,34]. The dynamic behavior of the simulation model of LCAD deficiency exhibits the highest accumulation of fatty acids (179-fold of C14 as can be seen in Figure 4) along with marked increase of these substrates during fasting (Figure 5 and Table 4) and the lowest production rate of acetyl-CoA (Figure 7). These findings might confirm gestational loss to be the explanation that no human cases of LCAD deficiency have been described.

Our model can be extended to comprehensively test and study deficiencies of mitochondrial trifunctional protein and β-hydroxy-acyl-CoA dehydrogenase, or other diseases of fatty acid oxidation such as carnitine cycle, electron transfer and ketone synthesis defects. Furthermore, differences in the expression level of the enzymes in different cells and tissues and their consequences on the dynamical behavior of the β-oxidation can be investigated. Future work will incorporate the enzymatic steps for unsaturated fatty acids.

## Conclusion

In summary, this work provides a stimulating example for Systems Biology in the context of human disease revealing insights into dynamic properties of complex biochemical networks under the constraints of various disease conditions. As analytical technologies for global and targeted measurements mature, especially with regards to metabolites, new findings and hypothesis can be verified utilizing quantitative data. Furthermore, while mitochondrial deficiencies are often treated with metabolites to stimulate the enzyme activities, models will allow evaluation of the influences of metabolite treatments at the mitochondrial level, visualization of the dynamic behavior of the pathway and exploration of a hypothetical rationale of the treatment. In this respect, computational biology proves to be able to uncover insights, which can hardly be obtained from experimental data alone.

## Methods

### Model

The computational model for mitochondrial β-oxidation was based on a publicly available E-Cell2 [35] simulation model developed by Yugi and Tomita [21]. The latter is a computational model of mitochondrial metabolism representative of the entire organelle. We chose that model as a starting point for building our own model as this model is based on knowledge gathered from quantitative studies of the organelle since the 1960s by dozen of researches. It consists of 58 enzymatic reactions and 117 metabolites, representing the respiratory chain, the TCA cycle, the fatty acid β-oxidation and the inner-membrane transport system. Previously published enzyme kinetics studies in the literature were successfully integrated and packaged into a single large model. All the enzymatic reactions are represented by rate equations found in literature. Of the total of 471 kinetic parameters, 286 are quoted from articles, whereas the rest of the parameters are computationally estimated [21,36]. A supplementary document describing that computational model can be found at . This work offered a perfect starting point to construct a kinetic model for fatty acid metabolism with particular focus on the β-oxidation cycle and the objective to enhance our understanding of various deficiencies of acyl-CoA dehydrogenases.

In order to strike a balance between simplicity and complexity of our simulation model, we extracted the β-oxidation part of the mitochondrial model (reducing complexity), modified and completed it with respect to simulating mitochondrial fatty acid oxidation deficiencies. The β-oxidation cycle together with the metabolite transporting system originally comprised 8 different enzymatic reactions with 5 different reaction mechanisms. The modifications of the original model comprise three central parts that are essential for simulating mitochondrial fatty acid oxidation deficiencies: (i) extension to the oxidation of stearoyl-CoA (C18), arachidonoyl-CoA (C20) and behenoyl-CoA (C22) (ii) input of short, medium and long chain fatty acids entering the β-oxidation cycle (iii) modeling of acyl-CoA dehydrogenase using four enzymes classified by their fatty acid chain length specificity.

There is no evidence that the first three steps of the β-oxidation cycle of stearoyl-CoA (C18), arachidonoyl-CoA (C20) and behenoyl-CoA (C22) are different from the steps of the other acyl-CoAs. We used the same parameters for stearoyl-CoA (C18), arachidonoyl-CoA (C20) and behenoyl-CoA (C22) as well as for the other acyl-CoAs, and explored only the parameters of the oxoacyl-CoA thiolase – the fourth step of the β-oxidation cycle – in dependence on the carbon chain length. However, simulations revealed that the variation of these parameters with regard to carbon chain length had no impact on acyl-CoA concentrations (data are not shown). Therefore, we set the parameters to the same values as for palmitoyl-CoA (C16).

Short and medium chain fatty acids (C4 – C12) were assumed to enter the β-oxidation cycle directly, whereas long and very-long chain fatty acids (C14–C22) were entering the cycle via the carnitine transporting system. The input of short, medium and long chain fatty acids entering the β-oxidation cycle depends on the concentration of the fatty acids in the inter-membrane space. A higher input results in a higher output – the production of acetyl-CoA – as can be seen in Figure 7. We modeled the input as a source with constant levels of acyl CoAs entering the β-oxidation cycle and the output as a sink resulting in a constant level of acetyl CoA. The distribution of the fatty acids in the body depends on several conditions like fatty acid transport conditions, metabolic conditions (e.g., fasting or catabolic stress), ingestion, to name just a few examples. Since we are interested in the accumulation of acyl-CoAs and the formation of acetyl-CoA for various deficiencies of acyl-CoA dehydrogenase and the corresponding dynamic response to moderate increase of palmitoyl-CoA (C16), we estimated the input of the beta-oxidation with the objective to fit and reproduce the experimentally derived acylcarnitine data of MCAD deficiency.

The modeling of acyl-CoA dehydrogenase using four enzymes classified by their fatty acid chain length specificity is based on experimental findings shown by the distribution of the enzyme activity in Figure 2.

We treated the mitochondrial matrix and intermembrane-space free carnitines as well as the intermembrane-space free CoA and acylCoA as fixed parameters in order to guarantee constant input of acylCoA to the beta-oxidation cycle. We did not get any enlarged acylcarnitine pools in all our calculations. If we set the mitochondrial matrix and intermembrane-space free carnitines variable, very small changes of mitochondrial matrix and intermembrane-space free carnitines were found resulting in a little bit smaller flux of acylCoA entering the beta-oxidation cycle. These very small changes did not impact on our results (data not shown). The level of matrix CoA was set high and variation of multiple magnitudes of that level did not impact on our results (data not shown).

The final model consisted of 64 reactions with 91 compounds (36 with fixed values) and 301 parameters. The entire model at different simulation states in SBML format can be found in the Additional files. The users can directly open the SBML files in COPASI [37] or use other simulation software , and modify this model of β-oxidation at different disease states.

### Simulation

Starting with initial concentrations, steady-state values were calculated under acyl-CoA dehydrogenase activity conditions of healthy controls. Subsequently, that steady-state of the model was the starting point to calculate steady-state values under mitochondrial fatty acid oxidation deficiencies conditions. These calculations are summarized in simulation A. Results of simulation A were compared and validated with experimental data. Finally, the models obtained in simulation A were used in simulation B where dynamic responses with respect to catabolic stress were calculated.

The initial metabolite concentrations (see Additional file 1) are taken from the literature or set approximately to their Km values of the enzymes [21]. Simulations are performed by numerical integration of the rate equations using the simulation software COPASI 4.2 Build 22 [37]. The acyl-CoA dehydrogenase activity was set according to healthy controls shown in Figure 2B. The new steady-state values of all metabolites were obtained by simulating the concentrations over a one year period (see Additional file 2).

### Simulation A

Starting from the steady-state values of healthy controls, the acyl-CoA dehydrogenase activity was changed according to Figure 2B. Again, the new steady-state values of all metabolites for SCADD, MCADD, LCADD and VLCADD were obtained by simulating the concentrations over a one year period (see Additional files 3, 4, 5, 6). Results of these simulations are shown in Figure 3 and 4.

### Simulation B

In order to analyze the dynamic behavior of the model, we increased palmitoyl-CoA (C16) in the inter-membrane space by 20%, 30% and 40%. The dynamical behavior of a 20% increase for the control group as well as for the different acyl-CoA dehydrogenase deficiencies are shown in Figure 5, 6. In Table 4 ratios of specific acyl-CoA concentrations before and 30 days after 20%, 30%, and 40% increase of palmitoyl-CoA (C16) are given. Finally, dynamical behavior concerning acetyl-CoA production simulating 20% as well as 40% increase of C16 is shown in Figure 7. We have not found any direct physiological evidence for our chosen 20% to 40% increase of palmitoyl-CoA with regards to catabolic stress. Our choice was first based on an assumption that was subsequently supported by our analysis of the dynamical behavior of the β-oxidation model. The performed simulations reveal that higher increases of C16 do not change the characteristic of the production rate of acetyl-CoA during the first few hours. We are interested in exploring this time period in particular, since rapid progress to coma of patients during fasting occurs within 1–2 hours. Additionally a higher increase of C16 results in higher accumulation of acyl-CoAs as well as increases the time to attain the steady-state of the system.

### Validation and limitation

The three central modifications of the original model are based on findings from literature or from performed simulations.

Overall validation was given by relating the calculated variables of the model (the metabolites of the β-oxidation cycle) to experimental available data from two new-born screening programs in Europe and Australia (simulation A). The overall characteristics of the simulated accumulation of acyl-CoA show good agreement with experimental data and findings in the literature (Figure 3 and Figure 4).

A limitation of our work relates to the direct comparison of calculated and measured data. It has to be considered that experimental data of the (isolated) mitochondria are not available and that the available measured data are not only influenced by the respective subsystem (the beta-oxidation cycle), but also may reflect additional effects caused by other subsystems and disease conditions.

Further limitations comprise that only saturated fatty acids were considered and that the β-oxidation of unsaturated fatty acids and odd-numbered chains of carbon were not modeled. Furthermore, we did not include carbon chain length dependencies of the activity of enoyl-CoA hydratase, β-hydroxyacyl-CoA dehydrogenase and 3-ketoacyl-CoA thiolase.

## Authors' contributions

RMO participated in the design of the study, carried out the computational calculations and drafted the manuscript. IO participated in the design of the study and helped to draft the manuscript with respect to biochemical and medical issues. BT conceived of the study, and participated in its coordination. GS helped to draft the manuscript. KMW performed newborn screening data analysis and helped to draft the manuscript with respect to biochemical issues. AG participated in the design of the study, performed newborn screening data analysis and helped to draft the manuscript. All authors read and approved the final manuscript.

## Supplementary Material

Additional file 1 **Initial state healthy controls**. Computational model of healthy controls at initial state Click here for file

Additional file 2 **Steady state healthy controls**. Computational model of healthy controls at steady state Click here for file

Additional file 3 **Steady state SCAD deficiency**. model of SCAD deficiency at steady state Click here for file

Additional file 4 **Steady state MCAD deficiency**. Computational model of MCAD deficiency at steady state Click here for file

Additional file 5 **Steady state LCAD deficiency**. Computational model of LCAD deficiency at steady state Click here for file

Additional file 6 **Steady state VLCAD deficiency**. Computational model of VLCAD deficiency at steady state Click here for file
